# Fusion of a Short HA2-Derived Peptide Sequence to Cell-Penetrating Peptides Improves Cytosolic Uptake, but Enhances Cytotoxic Activity

**DOI:** 10.3390/ph2020049

**Published:** 2009-09-25

**Authors:** Ines Neundorf, Robert Rennert, Jan Hoyer, Franziska Schramm, Kristin Löbner, Igor Kitanovic, Stefan Wölfl

**Affiliations:** 1 Institut für Biochemie; Fakultät für Biowissenschaften, Pharmazie und Psychologie; Universität Leipzig, Brüderstr. 34, D-04103 Leipzig, Germany; 2 Institut für Pharmazie und Molekulare Biotechnologie, Ruprecht-Karls-Universität Heidelberg, Im Neuenheimerfeld 364, D-69120 Heidelberg, Germany

**Keywords:** membrane fusion, hemagglutinin, cell-penetrating peptides, CAP18, cytotoxicity, drug delivery

## Abstract

Cell-penetrating peptides (CPP) have become a widely used tool for efficient cargo delivery into cells. However, one limiting fact is their uptake by endocytosis causing the enclosure of the CPP-cargo construct within endosomes. One often used method to enhance the outflow into the cytosol is the fusion of endosome-disruptive peptide or protein sequences to CPP. But, until now, no studies exist investigating the effects of the fusion peptide to the cellular distribution, structural arrangements and cytotoxic behaviour of the CPP. In this study, we attached a short modified sequence of hemagglutinin subunit HA2 to different CPP and analysed the biologic activity of the new designed peptides. Interestingly, we observed an increased cytosolic distribution but also highly toxic activities in the micromolar range against several cell lines. Structural analysis revealed that attachment of the fusion peptide had profound implications on the whole conformation of the peptide, which might be responsible for membrane interaction and endosome disruption.

## 1. Introduction

In the past twenty years the use of cell-penetrating peptides (CPP) has evolved as an attractive tool to solve the problems of drug delivery [1,2,3]. Until now, a great variety of peptide vectors has been tested for their ability to transport molecules of different size and charge into the cell interior. Recent reports describe the internalisation of nanoparticles [4], oligonucleotides [5], proteins [6] and liposomes [7]. Penetratin, a segment of antennapedia (Antp) homeodomain from *Drosophila*, [8] short fragments of Tat protein (the transactivator of transcription from HIV-1) [9] and VP22 (from herpes simplex virus type-1 transcription factor) [10] are among the most intensively studied CPP. However, one limiting step in the use of CPP is their enclosure in vesicles after cellular uptake, since most of the CPP used internalise via endocytotic pathways. Therefore, endosomal escape techniques are needed to support the outflow of the carrier-cargo construct into the cytosol. One promising approach takes advantage of membrane fusion proteins or peptides. Generally, membrane fusion plays a crucial role in the uptake and outflow of molecules and is also an essential step in other biological processes such as virus invasion into cells, or the cytolytic action of toxins. Often the fusion reaction is triggered by fusion peptides, most of them consisting of a short fusion domain of about 30 amino acids that are expected to form amphipathic helices. Influenza virus hemagglutinin (HA) is a glycoprotein that is responsible for the fusion of the virus capsid with the host cell membrane [11]. Recently, the N-terminal 20 residues of the HA2 subunit were identified as the fusion peptide [12]. Different studies showed that the fusion reaction of HA2 analogues is due to a conformational change at low pH-values leading to the formation of amphiphilic helices [13,14,15]. Due to their activity to disrupt endosomal membranes *N*-terminal HA2 fragments have been used to release biologically active substances into the cytosol after endosomal uptake [16,17,18,19,20]. Recently, this method was found to be useful for the delivery of Tat-fused anti-cancer peptides whereby the activity of Tat-shepheridin conjugates was significantly increased by this technique [21]. However, to our knowledge, the influence of the fusogenic peptide sequence to the properties of the CPP has not been investigated yet.

The aim of this study was to elucidate the biologic activity of chimeric peptides consisting of a very short HA2-derived peptide sequence and cell-penetrating peptides. The idea was to create efficient delivery vehicles that can be easily synthesised by solid phase peptide synthesis (SPPS) techniques and that own membrane destabilisation activities for an improved endosomal escape. 

## 2. Results and Discussion

We investigated three CPP coming from different sources such as the Tat(48-60) peptide, a human calcitonin (hCT)-based peptide with a branched structure (hCT-(18-32)-k7) [22] and a new carrier peptide derived from an antimicrobial peptide family (for peptide sequences, see Table 1). Recently, antimicrobial peptides have also been considered to act like cell-penetrating peptides [23]. Due to their cationic character and amphipathic nature they are able to interact with the lipid bilayer components. Cationic antimicrobial peptides (CAMPs) are present in all living organisms and can rapidly inactivate bacterial or viral pathogens by binding to and permeabilising the membranes of the target microorganisms. The CAMP cathelicidin (CAP18) [24,25] was reported to bind lipopolysaccharide (LPS) [26] and recently, fragments of CAP18 were investigated for their antimicrobial activity. Here, highly cationic sequences were identified in the *C*-terminal region (named C18, corresponding to residues 106-125) exhibiting an amphipathic α-helical conformation possibly responsible for the antimicrobial activity [27]. Moreover, previous work has shown that cationic α-helical antimicrobial peptides act as useful vehicles for gene delivery applications [28]. In fact, taking into consideration the sequence similarity of the C18 fragment to the Tat(48-60) peptide, we reasoned that a slightly shorter analogue (corresponding to residues 106-121 of CAP18), named sC18, might fulfil the requirements of a “good” CPP, as well. 

**Table 1:** Names, sequences and molecular weights of the investigated peptides.

Peptide	Sequence	MW_calc._ [Da]	MW_exp._ [Da]
sC18	GLRKRLRKFRNKIKEK	2068.5	2069.9
hCT(18-32)-k7	KFHTFPQTAIGVGAP	3164.8	3165.1
	KKRKAPKKKRKFA┘		
Tat(48-60)	GRKKRRQRRRPPQ	1717.0	1718.3
N-E5L	*GLLEALAELLE*	1169.3	1170.4
N-E5L-sC18	*GLLEALAELLE*GLRKRLRKFRNKIKEK	3220.9	3221.2
N-E5L-hCT(18-32)-k7	*GLLEALAELLE*KFHTFPQTAIGVGAP	4317.2	4319.0
	KKRKAPKKKRKFA┘		
N-E5L-Tat(48-60)	*GLLEALAELLE*GRKKRRQRRRPPQ	2869.4	2869.9

All peptides are amidated at the C-terminus; for internalisation studies all peptides were labelled with 5(6)-carboxyfluorescein at the *N*-terminus of the N-E5L sequence.

**Figure 1:**
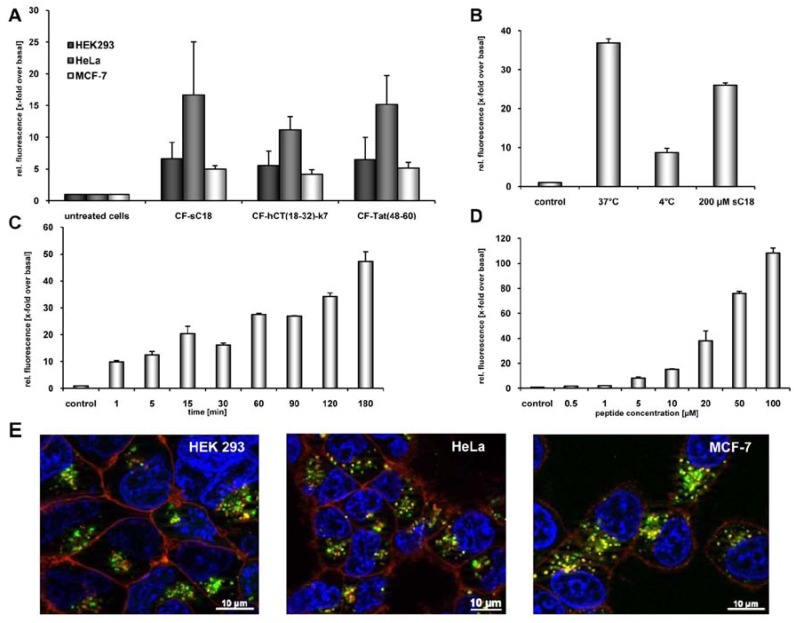
(a) Internalisation efficiencies of CF-labelled sC18, Tat(48-60) and hCT(18-32)-k7 in HEK 293, HeLa as well as MCF-7 cells, as confirmed by flow cytometry. Cells were incubated with 25 µM peptide solutions for 60 min. Subsequently, external fluorescence was quenched with trypan blue, cells were washed and detached. After washing and dilution with cold HBSS, cells were inspected. Each data point was done as triplicate counting at least 10,000 cells. (b) Internalisation of CF-sC18 in HEK293 cells as determined by flow cytometry. Uptake studies with 20 µM CF-sC18 at 37 °C and 4 °C and competition with 200 µM sC18. (c) And (d) time- (20 µM peptide solution) and concentration-dependence (60 min incubation time) of CF-sC18 uptake in HEK 293 cells. (e) Cellular uptake of 25 µM CF-sC18 within 60 min in HEK 293, HeLa and MCF-7 cells. Cells were counter-stained with transferrin-TexasRed, as marker for clathrin-dependent endocytosis, and the nuclei marker benzimide H33342.

We tested our hypothesis by first evaluating quantitatively the uptake of carboxyfluorescein (CF)-labelled sC18 by using flow cytometry. Therefore, HeLa, MCF-7 and HEK 293 cells, all of them often used model systems in terms of CPP drug delivery applications, were incubated for 60 min with 25 µM peptide solutions. As indicated in Figure 1A, CF-sC18 is a highly efficient cell-penetrating peptide compared to the other two investigated CPP. Furthermore, after incubation of HEK293 cells with the peptide solution at 4°C we observed a decreased fluorescence intensity (see, Figure 1B), which suggests an energy-dependent endocytotic uptake pathway. However, a small fraction seems to be able to enter cells also by direct cytosolic transfer. Moreover, a competition experiment (using an excess of unlabelled sC18) caused only minor decrease in uptake pointing to an uptake pathway independent of specific binding partners (e.g., receptor or transporter) (see, Figure 1B). In addition, incubating HEK293 cells for various time periods (Figure 1C) as well as with different peptide concentrations (Figure 1D) indicated that the uptake is both time- and concentration-dependent.

Furthermore, we investigated the subcellular localization of CF-sC18 by using fluorescence microscopy. HeLa, HEK 293 and MCF-7 cells were incubated for 60 min with 25 µM peptide solutions and inspected after quenching extracellular fluorescence with trypan blue solution. As expected from the former studies the peptides distributed in a vesicular pattern throughout the cytosol. As an initial exploration of the mechanism of uptake we co-treated the cells with transferrin-TexasRed, a marker for clathrin-dependent endocytosis. In the case of HeLa and MCF-7 cells co-localisation of peptide and marker within some vesicles was obvious (see, Figure 1E). However, we observed only minor merged fluorescence when incubating HEK293 cells with CF-sC18 and transferrin-TexasRed. Recently, Tat(48-58) was shown to enter cells mostly by macropinocytosis whereas for hCT(18-32)-k7 several endocytotic pathways have been suggested. [29,30] However, the obvious endosomal uptake hampers the function of all three CPP and thus, there is need of methods for enhancing the endosomal escape.

Therefore, we fused an analogue of the HA fusion-peptide from the *N*-terminal region of the HA2 subunit, named N-E5L, to the investigated CPP. This peptide is a truncated and modified version of the recently described E5L-peptide, an amphiphilic anionic peptide that is able to mimic the fusogenic activity of influenza HA [14]. All peptides were synthesised by standard Fmoc-*t*Bu SPPS (see Table 1). 

**Figure 2:**
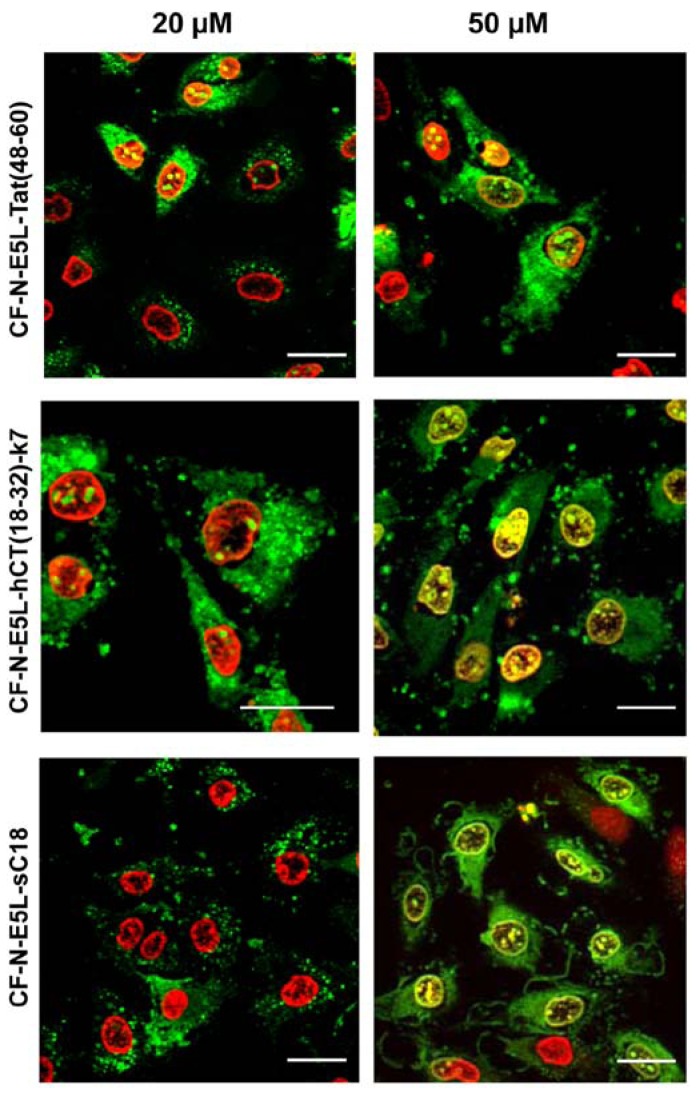
Cellular uptake of 20 µM and 50 µM CF-labelled chimeric N-E5L-CPP within 60 min in HeLa cells is shown. The cells were counter-stained with the nuclei marker benzimide H33342 that is illustrated in red for a clear display. External CF fluorescence was quenched with trypan blue solution. Scale bar is 20 µm.

At first, the internalisation activity of the new chimeric peptides was investigated in HeLa cells by fluorescence microscopy techniques. After incubating the cells with 20 µM peptide solutions both cells showing cytosolic as well as vesicular peptide distribution are visible. Interestingly, at higher concentrations (50 µM in Figure 2) all chimeric peptides also accumulated in the cell nucleus, and the morphology of the cells suggests partial destruction of the cell membrane. From the observation at 20 µM peptide concentrations one might conclude that the peptides are at first taken up by endocytosis and then released into the cytoplasma after an initial endo- and lysosome disruption, or, that they are taken up by direct cytosolic transfer. At higher concentrations the lytic activity of the peptides is not longer restricted to the membranes of the endolysosomal vesicles, but now also comprises the integrity of the nuclear membrane and the plasma membrane. Together with the liberated endolysosomal hydrolytic enzymes, a cascade of self-destruction is initiated, ultimately leading to cell death.

**Figure 3:**
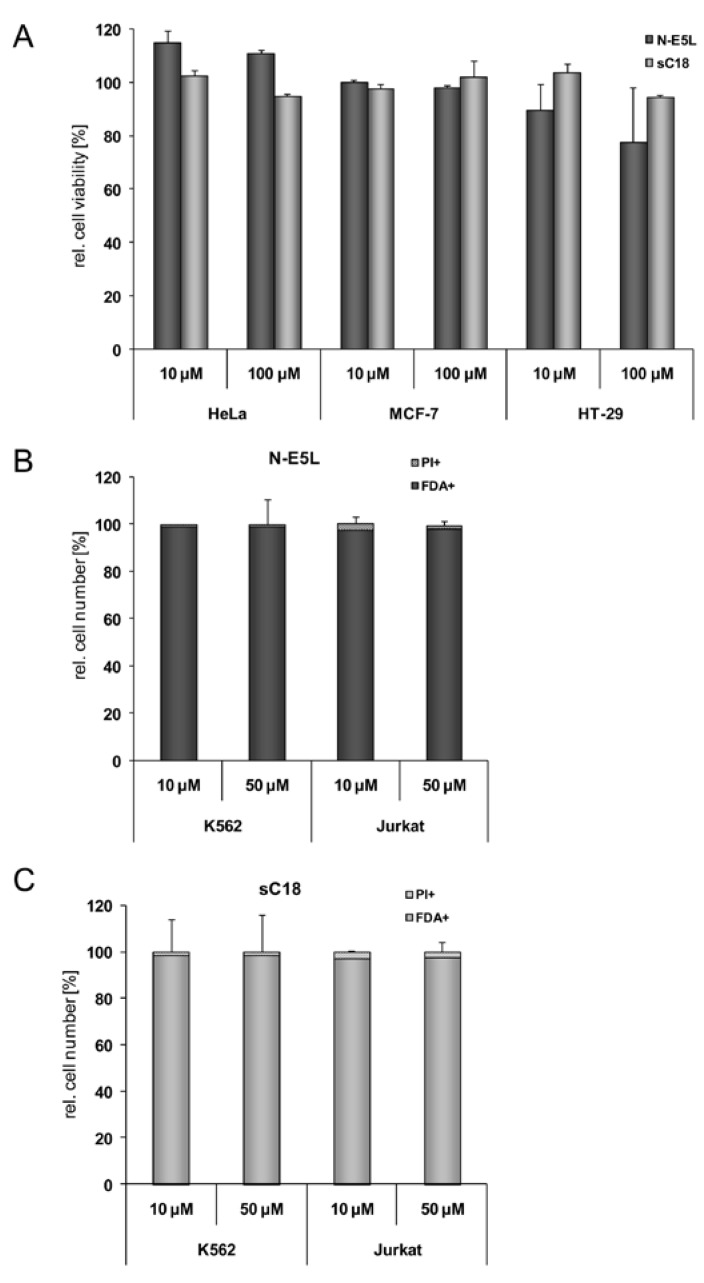
Cytotoxicity profile of sC18 and the N-E5L sequence in MCF-7, HT-29 and HEK293 cells after incubation with 10 µM and 100 µM peptide solutions (a). (b) N-E5L and sC18 (c) in K562 and Jurkat cells after incubation with 10 and 50 µM peptide solutions.

Therefore, we further elucidated the cytotoxicity profiles of the chimeric peptides for several cell lines (including MCF-7, HeLa and HT-29 cells, as well as suspension cell lines K562 and Jurkat). Recently, toxicity profiles for the CPP Tat(48-60) and hCT(18-32)-k7 have been reported and both peptides were assumed to be non-toxic within a defined concentration range in a series of tested cell lines [22,31]. Furthermore, it was described for Tat(48-60) that this peptide has no membrane toxicity in MDA-MB-231 breast cancer cells and no haemolytic effect to K562 erythrocytes [31]. Indeed, we could confirm these results in our cytotoxicity studies; no cytotoxic effects of Tat(48-60) and hCT(18-32)-k7 have been found for all tested cell lines (data not shown). However, we initially tested the influence of the parent CPP sC18 and the N-E5L sequences alone to several cell lines and also in this case, no cytotoxicity was observed within concentrations of up to 100 µM (see Figure 3A-C).

**Figure 4:**
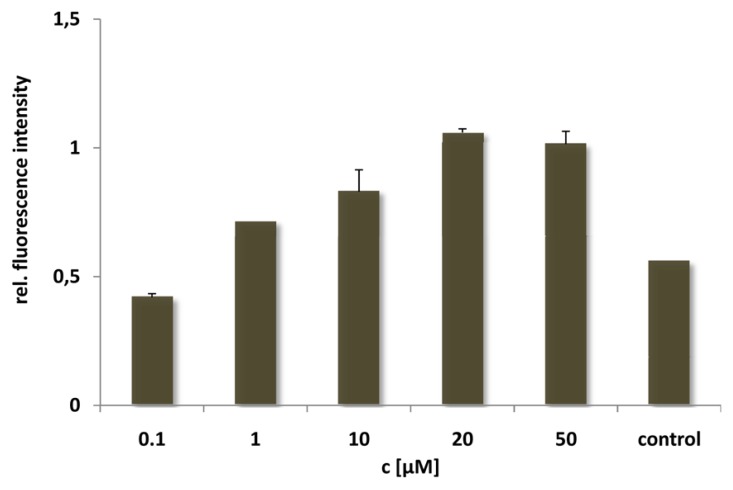
Cellular uptake of CF-labelled N-E5L after incubating HeLa cells for 60 min with peptide solutions of several concentrations. Untreated cells were measured as control.

For the N-E5L peptide this is not remarkable, since we found out that alone it is unable to translocate in cells. After incubation of the CF-labelled analogue we neither observed fluorescent cells microscopically nor by quantification using flow cytometry (see for example, Figure 4, after incubation of HeLa cells with CF-N-E5L).

**Table 2:** IC_50_ values of the N-E5L-peptides for the investigated cell lines.

Peptide	IC_50_ [µM]				
	HeLa	MCF-7	HT-29	Jurkat	K562
N-E5L-sC18	31.8 ± 1.9	30.8 ± 4.1	72.1 ± 0.1	7.3 ± 1.7	> 30
N-E5L-hCT(18-32)-k7	33.1 ± 3.3	37.8 ± 6.5	59.1 ± 0.1	11.4 ± 4.2	> 30
N-E5L-Tat(48-60)	29.4 ± 3.9	39.3 ± 9.4	90.9 ± 0.2	7.9 ± 0.8	> 30

As indicated in Table 2, the IC_50_ values of all chimeric peptides were in the lower micromolar range. The adherent cell lines HeLa and MCF-7 turned out to be more sensitive than HT-29 cells. This is probably due to our observation that HT-29 cells do not internalise CPP with the same efficiency as HeLa and MCF-7 cells (unpublished data). In comparison, Jurkat cells seem to be even more sensitive to the cytotoxic peptides. This is not too unexpected, concerning the fact that suspension cells often are able to take up higher amounts of substance due to better surface exposure. Interestingly, the IC_50_ values are in the same concentration range for all chimeric CPP constructs. Potentially, this can be explained by nearly the same uptake rate into the different cell lines tested. Indeed, as indicated in Figure 1A, it seems that all tested peptides have similar internalisation efficiencies. In addition, cell viability for HeLa cells was in the range of 90-95% at a concentration of 20 µM. Therefore, we conclude that the observed increased cytosolic distribution of the peptides at this concentration might be the result of a better escape out of the endosomes.

**Figure 5:**
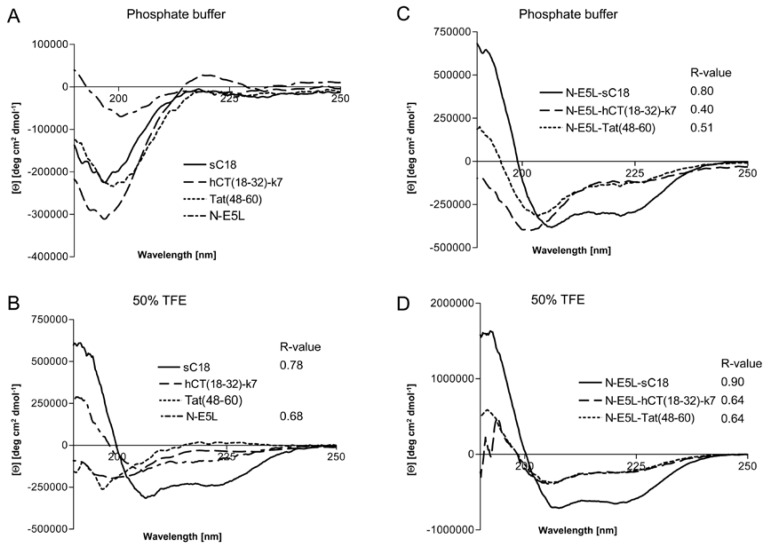
Circular dichroism spectra of the investigated peptides. The spectra were measured at a peptide concentration of 20 µM in 10 mM phosphate buffer [(a), (c)] and 10 mM phosphate buffer with the addition of 50% TFE [(b), (d)]. R-values represent the ratios between the molar ellipticity values at 222 nm and 207 nm.

However, to get some more insights into the structural arrangements of the investigated peptides and thus, the possible mode of action we performed CD spectroscopy measurements. We dissolved the peptides in 10 mM phosphate buffer as well as in phosphate buffer supplemented with trifluorethanol (TFE), a helix inducing solvent. All parent CPP as well as the N-E5L peptide alone display a spectrum typical of an unordered conformation in 10 mM phosphate buffer, pH 7 (Figure 5A), with minima around 197 nm – 200 nm. At greater than 50% TFE, the shape of the spectrum of sC18 and N-E5L, with minima around 208 nm and 222 nm, indicated that it had acquired a significant level of α-helical conformation, whereas, Tat(48-60) and hCT(18-32)-k7 are still in an unstructured conformation with minima of 197 nm and 200 nm, respectively (Figure 5B). For the peptide hCT(18-32)-k7 this is in agreement with recently published results [32]. Recently, also for the parent fragment C18 an α-helical structure was reported [27]. In contrast, after attachment of the fusogenic sequence N-E5L to sC18 the spectra displayed α-helical structures both in phosphate buffer as well as in the presence of TFE compared to N-E5L-hCT(18-32)-k7 and N-E5L-Tat(48-60) where the α-helical content is already present in phosphate buffer alone but more obvious after the addition of TFE (Figure 5C and Figure 5D). 

Apparently, the attachment of the HA2 peptide fragment to the CPP supports the formation of helical structures. This was further supported after calculating the ratio between the molar ellipticity at 222 nm and 207 nm *R* = [Θ]_222_/[Θ]_207_, a useful parameter for the interpretation of CD spectra [33]. It has been demonstrated that this value is approximately 1 for α-helices, while it decreases to 0.4 in 3_10_ helices [34]. As seen from Figure 3 the *R*-values become significantly higher for the N-E5L peptides and support an increased content of α-helical conformations for the chimeric peptides. Since the formation of amphiphilic helixes is often responsible for membrane-participating processes [35,36], we assume that the observed α-helical conformations are involved in the membrane permeabilizing activity of the chimeric peptides. The determined cytotoxic effects could be on the one hand the result of direct disruption of the cellular membrane and on the other hand the consequences of endosome disruption.

**Figure 6:**
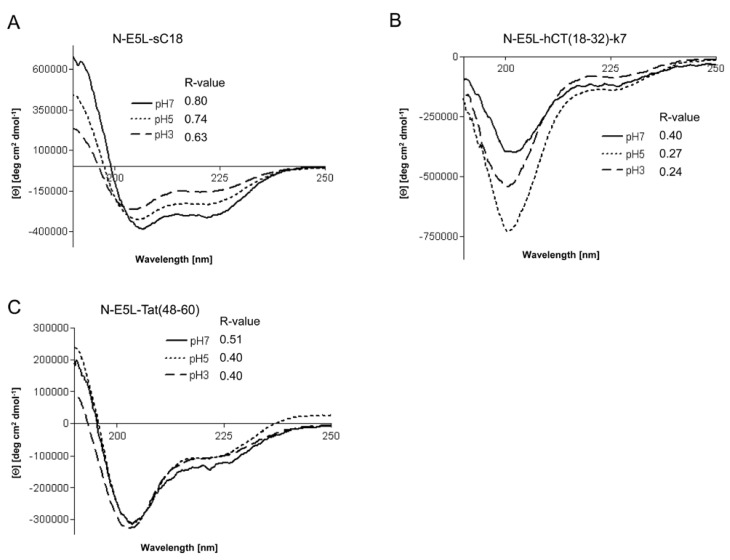
Circular dichroism spectra of N-E5L-sC18 (a), N-E5L-hCT(18-32)-7 (b) and N-E5L-Tat(48-60) (c) at different pH values. The spectra were measured at a peptide concentration of 20 µM in 10 mM phosphate buffer. R-values represent the ratios between the molar ellipticity values at 222 nm and 207 nm.

However, recently it was demonstrated for fusion peptide analogues of HA2 that they contain higher α-helical contents at acidic pH [15]. We therefore determined whether the investigated chimeric peptides undergo a conformational change under acidic pH that might be responsible for endosome disruption. Interestingly, the CD spectra at pH 3 and pH 5 revealed that all peptides had also assumed an α-helical conformation at acidic pH, as shown in Figure 6. Moreover, the α-helical content seem to be slightly less than at neutral pH (as assumed by the determined R-values), however, the differences at lower pH seem not to be enough to be considered important. 

**Figure 7:**
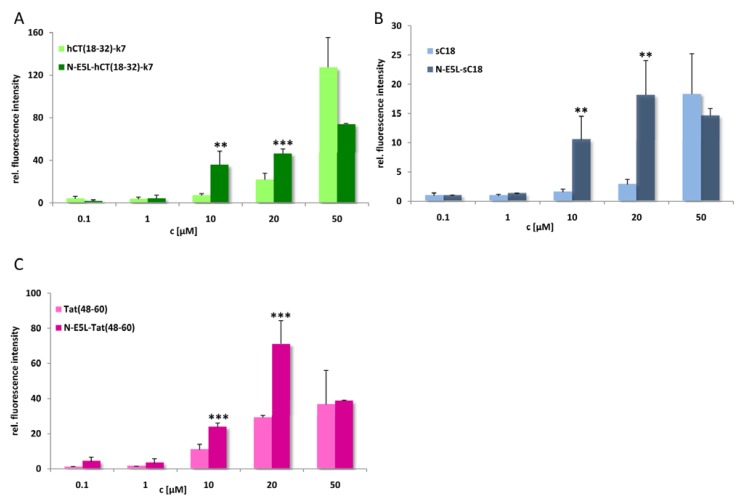
Cellular uptake of CF-labelled hCT(18-32)-k7 and N-E5L-hCT(18-32)-k7 (a), sC18 and N-E5L-sC18 (b), and Tat(48-60) and N-E5L-Tat(48-60) (c) after incubation of HeLa cells 10 min with several peptide concentrations. Modification with the N-E5L peptide leads to higher uptake values. At concentrations > 30 µM a cytotoxic influence of the chimeric peptides is observed. (Values were compared by using student’s unpaired t-test, p < 0.01**; p < 0.001***).

Thus we conclude that highly stable α-helixes are already formed at pH 7, that are responsible for the membrane-interacting processes. At the moment, we cannot explain if the observed cytosolic distribution is a result of endosomal escape or direct cytosolic transfer. However, after quantifying the amount of internalised chimeric peptide after 10 min in HeLa cells we observed that the uptake is more efficient and faster (see Figure 7). Moreover, since, as already mentioned, up to a concentration of 20 µM cell viability for HeLa cells was still in the range of 90-95% the observed increased cytosolic uptake at this concentration gives a hint that the peptides are indeed released more efficiently out of the endosomes. Therefore, further studies are now under investigation to elucidate drug delivery efficiency at minor concentrations to circumvent the observed toxic influence on cell viability and to increase the biologic activity of the cargo.

## 3. Experimental

### 3.1. Materials

N^α^-Fmoc-protected amino acids were purchased from IRIS Biotech (Marktredwitz, Germany), 1-hydroxybenzotriazole (HOBt) and 4-(2’,4’-dimethoxyphenyl-Fmoc-aminomethyl)phenoxy (Rink amide) resin were obtained from NovaBiochem (Läufelfingen, Switzerland), diisopropylcarbodiimide (DIC), the resazurin-based *in vitro* toxicology assay kit, 3-(4,5-dimethylthiazol-2-yl)-2,5-diphenyltetrazolium bromide (MTT), propidium iodide (PI) from Sigma-Aldrich (Taufkirchen, Germany), trifluoroacetic acid (peptide synthesis grade) from Riedel-de Haën (Seelze, Germany). *O*-(7-azabenzotriazol-1-yl)-1,1,3,3-tetramethyluronium hexafluoro-phosphate (HATU), *N,N*-diisopropyl-ethylamine (DIEA), thioanisole, p-thiocresole, piperidine, trifluoroacetic acid (HPLC grade), trypan blue and 5(6)-carboxyfluorescein (CF) were purchased from Fluka (Taufkirchen, Germany). Bisbenzimide H33342, dihydroethidium (D1168) and fluorescein diacetate (FDA) were obtained from Invitrogen (Karlsruhe, Germany). *N*,*N*-dimethylformamide, dichloromethane and diethyl ether were from Biosolve (Valkenswaard, Netherlands). Acetonitrile (ACN) was from Merck (Darmstadt, Germany). The following side chain protecting groups were used: *tert*-butyl (*t*Bu) for Thr; *tert*-butyloxy (*t*BuO) for Glu; trityl (Trt) for Asn, Gln and His; 2,2,4,6,7-pentamethyl-dihydrobenzofurane-6-sulfonyl (Pbf) for Arg; *tert*-Butyloxycarbonyl (Boc) and 1-(4,4-dimethyl-2,6-dioxocyclohex-1-ylidene)ethyl (Dde) for Lys. The following cell media and supplements were used: Dulbecco’s modified eagle medium (DMEM) and Ham’s F12 (without L-glutamine), RPMI 1640 (with L-glutamine), OptiMEM, HBSS, Dulbecco’s phosphate buffered saline (PBS) without calcium and magnesium, foetal calf serum (FCS), bovine serum albumin (BSA) and trypsin/EDTA (0.05%/0.02% in PBS) were obtained from PAA (Cölbe, Germany). Cell culture flasks (75 cm^2^) and 96-well plates were from TPP (Trasadingen, Switzerland). Glass-bottomed 8-well plates used for fluorescence microscopy studies were from ibidi GmbH (Martinsried, Germany). Fluoromount-G was obtained from SouthernBiotech (Birmingham, UK).

### 3.2. Peptide synthesis

All peptides were prepared by using an automated multiple solid-phase peptide synthesiser (Syro, MultiSynTech, Bochum, Germany) according to the Fmoc-*t*Bu strategy as described elsewhere [37]. The peptides were synthesised as *C*-terminal amides using the Rink amide resin (0.5 mmol peptide per gram resin). The branched hCT-derived peptide was prepared as described previously [22]. To allow microscopical and spectroscopical detection all peptides were *N*-terminally labelled with carboxyfluorescein (CF). The labelling was performed for 1 h at room temperature in DMF by using a 1.5-fold excess of CF, DIEA and HATU while still bound to the resin with fully protected side chains. Finally the peptide amides were cleaved from the resins with TFA:thioanisole:thiocresole (90:5:5, v/v/v) within 3 hours at room temperature, removing all acid-labile protecting groups simultaneously. After precipitation from and five times washing with ice cold diethyl ether, the peptides were collected by centrifugation, lyophilised from water:*tert*-butyl alcohol (3:1 v/v), and analysed by analytical RP-HPLC on a Vydac RP18-column (4.6 × 250 mm; 5 µm/300 Å) using linear gradients of 10-60% B in A (A = 0.1% TFA in water; B = 0.08% TFA in acetonitrile) over 30 min and a flow rate of 0.6 mL min^−1^. Further purification of the peptides was achieved by preparative HPLC on RP18 column (Waters, 5 µm, 25 × 300 mm) by using a linear gradient of 20-50% B in A over 50 min and a flow rate of 15 mL min^−1^. All peptides were obtained with purities >95%. Identification was performed by MALDI-ToF-ToF mass spectrometry (Ultraflex III ToF/ToF, Bruker Daltonics). The peptide sequences are given in Table 1.

### 3.3. Cell culture

We used the following cell lines: HEK 293 (human embryonal kidney), HeLa (human cervix carcinoma), MCF-7 (human breast adenocarcinoma), HT-29 (human colon adenocarcinoma), K-562 (human chronic myeloid leukemia) and Jurkat (human T cell leukemia). All cells were cultured in 75 cm^2^ culture flasks at 37 °C and 5% CO_2_ in a humidified atmosphere and split when confluent. For HEK 293 cells Dulbecco’s Modified Eagle Medium/ Ham’s F12 (without L-glutamine) containing 15% heat-inactivated foetal calf serum was used. HeLa and Jurkat cells were grown in supplemented RPMI 1640 (with L-glutamine) with 10% heat-inactivated foetal calf serum, MCF-7 cells in Dulbecco’s Modified Eagle Medium/ Ham’s F12 (without L-glutamine) containing 10% heat-inactivated foetal calf serum and 1% glutamine, and HT-29 and K-562 cells in Dulbecco’s Modified Eagle Medium supplemented with 10% heat-inactivated foetal calf serum. Detachment and dissociation of confluent cells was done by Trp/EDTA (0.05%/0.02% in PBS). For microscopic studies cells were seeded in glass-bottomed 8-well plates, whereas cells for cell viability and flow cytometric studies were cultured in 96-well plates.

### 3.4. Microscopy studies of peptide uptake

The cellular peptide uptake was investigated microscopically with HEK 293, MCF-7 and HeLa cells, seeded on glass-bottomed 8-well plates (ibidi, München). When grown to subconfluency, the cells were incubated with 20 µM, 25 µM and 50 µM, respectively, of the CF-labelled peptides in OptiMEM at 37 °C for 1-2 h. To counter-stain the cells with transferrin-TexasRed for clathrin-dependent endocytosis, cells were incubated with 50 µg/mL dye solution for 90 min during the peptide incubation. The nuclei were stained for 30 min with 2.5 µg benzimide H 33342 for 30 min prior to the end of the peptide incubation. Subsequently, the peptide solution was removed and external CF fluorescence was quenched by treating the cells for 1 min with trypan blue (6.5 mM in sodium acetate buffer, pH 4.5) [38]. Finally, cells were washed twice with HBSS and investigated by using a Zeiss Axiovert 200 inverted fluorescence microscope with ApoTome.

### 3.5. Resazurin/MTT-based cell viability assay

The effect of the peptides on the viability of HeLa cells was examined by using a resazurin-based *in vitro* toxicology assay kit. Cells were grown to subconfluency in 96-well plates and then incubated for 24 h under standard growth conditions with up to 100 µM peptide solutions in cell culture medium. As negative and positive controls untreated cells and cells treated 10 min with 70% EtOH were used. Following the incubation the cells were washed with medium without foetal calf serum and subsequently incubated at 37 °C for 2 h with a 10% solution of resazurin in medium without foetal calf serum. Finally, the conversion of resazurin to the reduced resorufin was measured fluorometrically at 595 nm (excitation at 550 nm) with a Spectrafluor plus multiwell reader (Tecan, Crailsheim, Germany).

The effect of the peptides on the viability of HT-29 and MCF-7 cells was checked by using the MTT-assay. Therefore, cells were cultivated to subconfluency under standard conditions in 96-well plates. The treatment was performed with 1-100 µM of the peptides for 24 h. Then, 10-fold stock solution of MTT reagent (5 mg/mL in medium) was added to each sample, and the cells were incubated for further 2 h. Formazan crystals were solubilised with 200 µL DMSO per well. After incubation for 15 min at 400 rpm and RT, absorbance was measured with a Tecan Saphire plate reader (560 nm, reference filter 630 nm).

### 3.6. Flow cytometric analysis of Jurkat and K562 cell viability

The viability after peptide treatment (1-50 µM peptide solutions) for 24 h of the suspension cell lines Jurkat and K562 was tested by using flow cytometry. For this purpose, 2.5 × 10^5^ cells per sample were stained with fluorescein diacetate (FDA) (0.04 µg/100 µL cell suspension) and propidium iodide (PI) (25 µg/100 µL cell suspension) in FACS buffer (1% BSA in PBS). Samples were incubated for 15 min at RT in the dark, washed and resuspended in FACS buffer.

Viability of the stained cells was analysed using a FACS^®^Calibur and CellQuest Pro analysis software (both Becton Dickinson, Franklin Lakes, USA) and is represented as relative cell number of viable cells. Untreated cells were used as negative control. Excitation and emission settings were 488 nm and 525–550 nm for FDA and 488 nm and 564–606 nm for PI staining.

### 3.7. Flow cytometric studies of peptide uptake

The cellular peptide internalisation was quantified by using a CyFlow ML flow cytometer (Partec, Münster, Germany). Initially, we used HEK 293 cells to examine the dependence of the cellular uptake of CF-sC18 on the peptide concentration, the incubation time and the temperature as well as its competition with a 10-fold excess of unlabelled peptide. In case of the concentration dependence we used several concentrations of CF-sC18 up to 100 µM, whereas the influences of incubation time, temperature and excess of unlabelled peptide where studied with 20 µM CF-sC18.

For this purpose, cell media where displaced by accordant peptide solutions in OptiMEM. After an incubation of 60 min – in case of the time dependence 1 min – 180 min – at 37 °C the peptide solution was removed and external CF fluorescence was quenched by a 1 min treatment with trypan blue (0.13 mM in sodium acetate buffer; pH 4.4). The cells were rinsed with OptiMEM and HBSS, respectively, detached with Trp/EDTA (0.05% / 0.02% in PBS) and resuspended in cold HEK 293 cell culture medium. For the flow cytometric measurement each sample was diluted with 600 µL cold HBSS.

Additionally, the CF-sC18 internalisation was investigated in HEK 293, HeLa and MCF-7 cells. A 25 µM peptide solution in OptiMEM was prepared and incubated with the cells for 60 min at 37 °C. External fluorescence was quenched with trypan blue, the cells were washed, detached and resuspended in the accordant cell medium as described above. Finally, cells were diluted with cold HBSS and inspected. Excitation and emission settings were 488 nm and 510 ± 10 nm. Each data point was done as triplicate counting at least 10,000 cells.

### 3.8. CD spectroscopy

CD spectra were recorded from 250 nm to 190 nm at 20 °C using a Jasco J-715 spectropolarimeter purged with N_2_ gas. Peptide samples were dissolved in 10 mM sodium phosphate buffer (pH 7) containing 0 or 50% (v/v) TFE. For measuring CD spectra at different pH, the buffer solution was set to pH 3 or pH 5, the peptides were dissolved to a final concentration of 20 µM and the pH was additionally controlled. The CD spectra were recorded for all solutions at a final peptide concentration of 20 µM. Each measurement was repeated three times using a sample cell with a path length of 0.2 cm. Instrument parameters were: response time 2 s, scan speed, 50 nm/min, sensitivity, 20 mdeg, step resolution, 0.2 nm and bandwidth, 1 nm. All CD spectra were corrected by substraction of the CD spectrum of the solvent to eliminate the interference from cell, solvent and optical equipment. The ellipticity was expressed as molar ellipticity [Θ] in deg cm^2^ dmol^−1^.

## 4. Conclusions

In conclusion, this study presents the design and characterisation of chimeric peptides consisting of a short fusogenic peptide sequence covalently linked to a cell-penetrating peptide moiety. In this regard we introduced a new very efficient CPP, a shortened form derived from the antimicrobial peptide CAP18, which we named sC18. This CPP is internalised by an endocytotic uptake pathway to nearly the same efficiency as Tat(48-60) and the human calcitonin-derived peptide hCT(18-32)-k7. Currently, we are studying the application of this carrier peptide as delivery vehicle for several different cargoes as organometallic complexes, proteins and siRNA. However, the novel designed chimeric peptides turned out to have an increased cytosolic distribution but intrinsic strong cytotoxic activities against certain cell lines. CD spectra of the chimeric peptides proved that by the attachment of the fusogenic peptide sequence α-helical arrangements are favoured. We propose that this is one important prerequisite for the overall membrane-disruptive activity that leads to endosomal escape and higher uptake values, as well. Furthermore, in combination with other anti-cancer and anti-leukemia drugs, these peptides could provide a significantly improved anti-cancer activity over established protocols. In particular it may be possible that anti-cancer drugs can be directly linked to these peptides leading not only to improved uptake of the drug but also increased cytotoxicity mediated directly by the peptide. In addition to our results observed in the treatment of human cell lines, we are although interested, if these peptides are active on bacterial membranes, which is also an aspect of our studies and is now currently under investigation. 
